# Incarcerated Rectal Prolapse Secondary to Rectal Adenomatous Polyposis: A Case Report

**DOI:** 10.7759/cureus.108985

**Published:** 2026-05-16

**Authors:** Alexandra L King, Raymond I Okeke, Katelynn Montgomery, Farrell Landwehr, Eugene Ismailov, John Underwood, Erik Grossman, Salman Ahmad

**Affiliations:** 1 Department of Surgery, University of Missouri School of Medicine, Columbia, USA

**Keywords:** adenoma, constipation, incarcerated prolapse, polyposis, prolapse, prolapsed rectal mass, rectal polyps, rectal prolapse, strangulated prolapse, villous adenoma

## Abstract

Incarcerated or strangulated prolapsed rectal polyps are an incredibly rare phenomenon, seldom reported in the literature. Given the overall low incidence of rectal prolapse, these instances in which polyposis - a relatively common condition - progress and become emergent are clinically significant. We present a case of a 49-year-old with an acutely incarcerated rectal prolapse of dysplastic villous adenomatous polypoid tissue. The patient presented to our facility with a large, painful rectal mass, passing mucus and bright red blood per rectum after two hours of straining to have a bowel movement. She denied past colonoscopies, anal receptive intercourse, and any personal or family history of bowel disease or cancer. On computed tomography (CT) scans, the incarcerated mass appeared as a full-thickness rectal prolapse with signs of edema and ischemia, prompting emergent surgical intervention. A diagnostic laparoscopy showed no pathology to the large bowel or rectum. The prolapsed mucosal mass was circumferentially resected, and the rectum was reduced. A temporary diverting loop sigmoid colostomy was then created. Surgical pathology reported villous adenoma, extensive high-grade dysplasia, and foci of pseudo-invasion with no evidence of invasive carcinoma. A follow-up rectal exam under anesthesia and flexible sigmoidoscopy identified multiple large rectal polyps but no evidence of rectal ischemia. The patient had an uneventful postoperative course and was discharged home on hospital day 11 for outpatient follow-up with Colorectal Surgery. Abdominal perineal resection versus transanal transabdominal rectal resection, preserving gastrointestinal continuity, have been offered as options for definitive treatment. Rectal prolapse can be predisposed to occur in the setting of a variety of clinical conditions. Management of prolapses can be complex. In the setting of these rare incarcerated or strangulated prolapsed dysplastic polyps, emergent surgical intervention is still indicated.

## Introduction

Rectal adenomas and rectal polyps are relatively common. However, their prolapse through the anal canal has scarcely been reported in the literature. Instances of rectal prolapse of any kind are a rare phenomenon estimated to affect less than 0.5% of the U.S. population, though exact incidence is unknown [1,2]. Even further, presentations of either incarcerated or strangulated rectal prolapses have seldom been described. Although the exact incidence of rectal prolapse due to polyposis is unknown, rectal prolapse overall is most often associated with predisposing anatomic abnormalities, generalized weakness of the pelvic floor, and concomitant prolapse of the uterus, vagina, or bladder organ [2]. Emergent presentations of rectal prolapse, in particular those without a clear predisposing factor, are unique. Rectal prolapse of any cause is an unusual condition, however prolapse due to polyposis, as well as development into incarceration, are both further remarkable features.

## Case presentation

**Figure 1:**
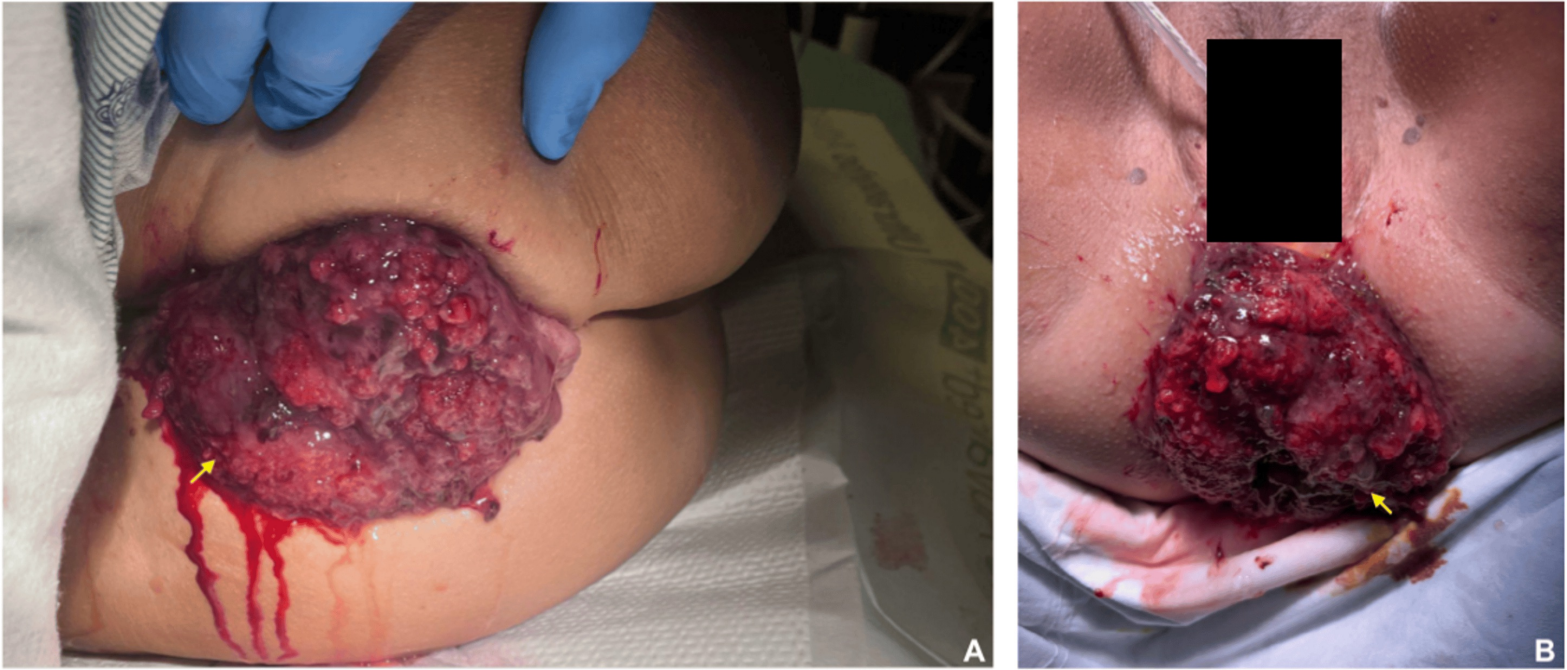
Preoperative images of 12.7 cm (5 in) exposed protrusion of giant rectal adenoma (yellow arrows). (A) Posterior view of prolapsed rectal tissue. (B) Anterior view of prolapsed rectal tissue.

A 49-year-old woman presented to our emergency department as a transfer from an outside facility, with a large mass protruding from her anus. She had been straining to have a bowel movement for two hours. She presented with perineal discomfort and pain, though she denied symptoms of nausea or vomiting. The patient reported intermittent periods of constipation that had been ongoing for the past few years. She has no history of gastrointestinal disease, cancer, or anorectal receptive intercourse. She denied any family history of bowel disease or cancer. Past surgical history was significant for one cesarean section, as well as a partial hysterectomy with removal of a benign mass. She had never had a colonoscopy. She was hemodynamically stable at initial presentation. The rectal mass was friable, ovoid, with a micro-lobulated, irregular surface. It was notably hemorrhagic with bright red blood, and the protruding portion was 12.7 cm (5 inches) in diameter (Figure 1). On digital rectal exam, no stool was noted, and no evidence of obstruction was found. Labs were significant for anemia (Hgb = 9.1 g/dL [Reference range = 12-16 g/dL]), leukocytosis (WBC = 22 x 109/L [Reference range = 5-10 x 109/L]), and hypokalemia (3.1 mmol/L [Reference range = 3.5 - 5.0 mmol/L]). The patient received I.V. potassium for repletion and ertapenem while in the E.D. She was transfused with 2 units of packed red blood cells and 2 units of fresh-frozen plasma.

**Figure 2:**
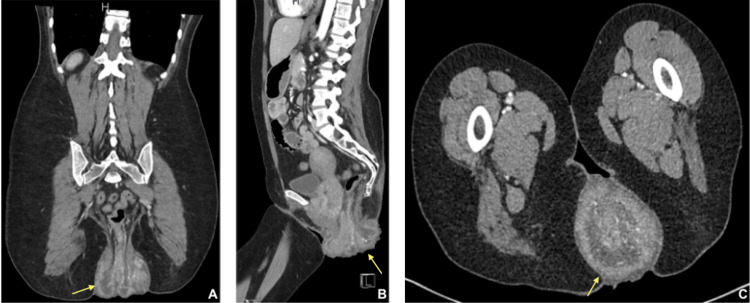
Preoperative computed tomography imaging with contrast, demonstrating a full-thickness, hyperdense rectal prolapse (yellow arrows), with edema and signs of ischemia. (A) Coronal view. (B) Sagittal view. (C) Transverse view.

The patient had a complete external full-thickness rectal prolapse that could not be manually reduced even after the application of sugar. CT imaging demonstrated a fluid-filled, hyperdense, edematous mass (Figure 2). Given this incarceration and concern for ischemia, the patient was booked for emergent rectal examination under anesthesia, possible colonoscopy, diagnostic laparoscopy with possible bowel resection or ostomy creation, and admitted to the Acute Care Surgery service. The patient was in the operating room within two hours of arriving at our emergency department.

In the operating room, a digital rectal exam under anesthesia revealed large, friable, and bleeding incarcerated rectal mucosal lesions. The rectal lumen was easily identified, without evidence of obstruction or stool burden. Manual reduction was unsuccessful, therefore a partial resection was performed. Using a perineal approach, the rectal mass, now comprising large mucosal lesions and external hemorrhoidal tissue, was resected circumferentially. A Bovie and an ENSEAL energy device (Ethicon, Inc., Raritan, NJ, USA) were used for resection, as well as 2.0 silk suture ligatures for dissection. Completion of resection resulted in spontaneous reduction of the remaining tissue. Digital exam confirmed patency, hemostasis, and a normal rectal tone. The rectum was then packed with a piece of SURGICEL SNoW within a SURGICEL sheet (Ethicon, Inc.) as a hemostatic gauze and secured with an abdominal pad and mesh underwear.

Diagnostic laparoscopy revealed an unremarkable sigmoid colon and rectum above the peritoneal reflection. No tethering or tension was appreciated. The proximal and distal ends of the sigmoid colon were held, and orientation was confirmed using a colonoscope inserted through the rectum. The sigmoid colon was then externalized as a loop through a circular incision in the left lower quadrant of the abdomen. A red rubber catheter was placed through a mesenteric defect as an ostomy bridge, and a transverse colotomy was made. An ostomy appliance was applied after maturing and closing the incisions, and a temporary diverting loop sigmoid colostomy was created. 

**Figure 3:**
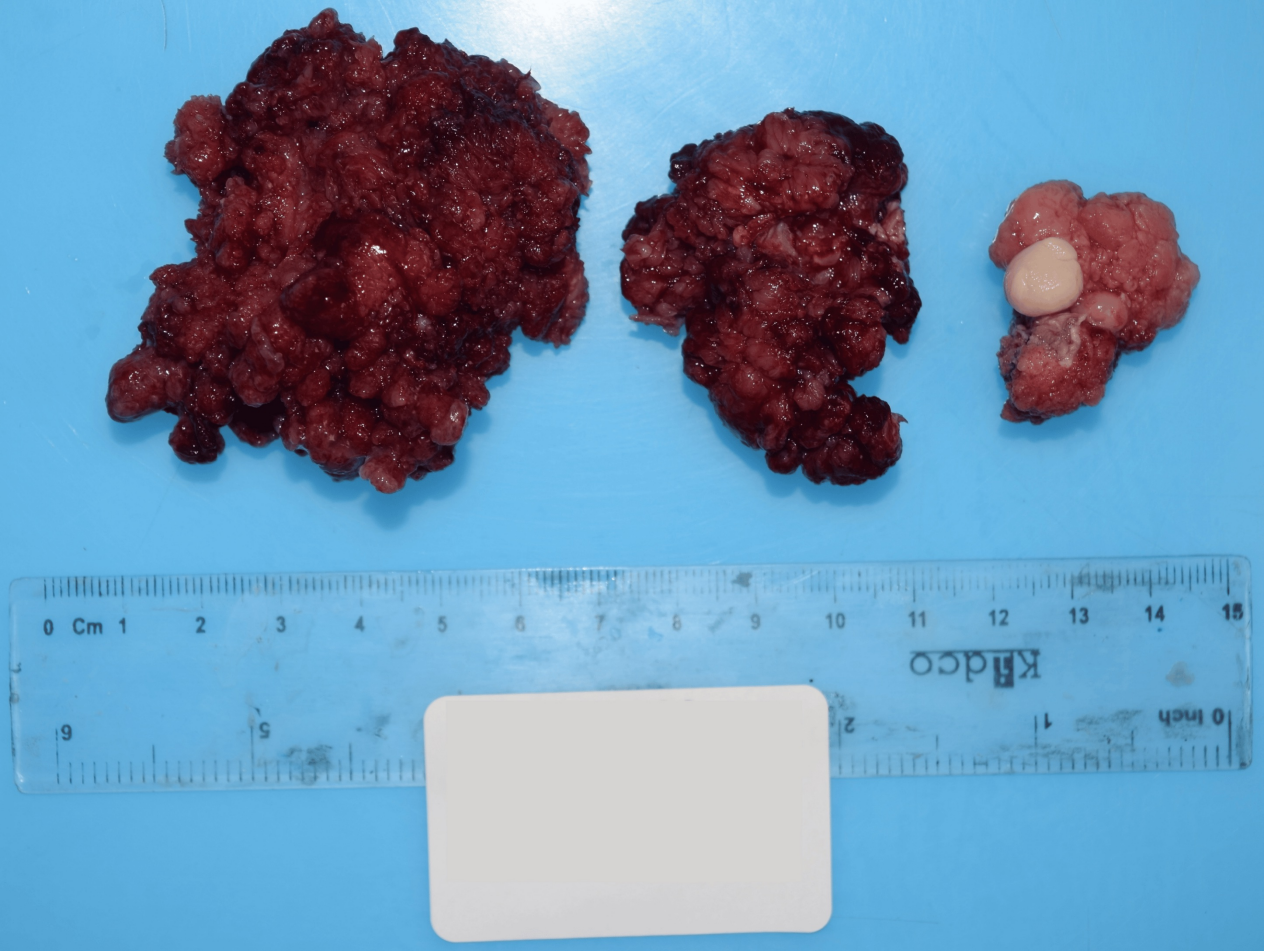
Gross image of resected specimen, an aggregate of polyploid mucosa.

**Figure 4:**
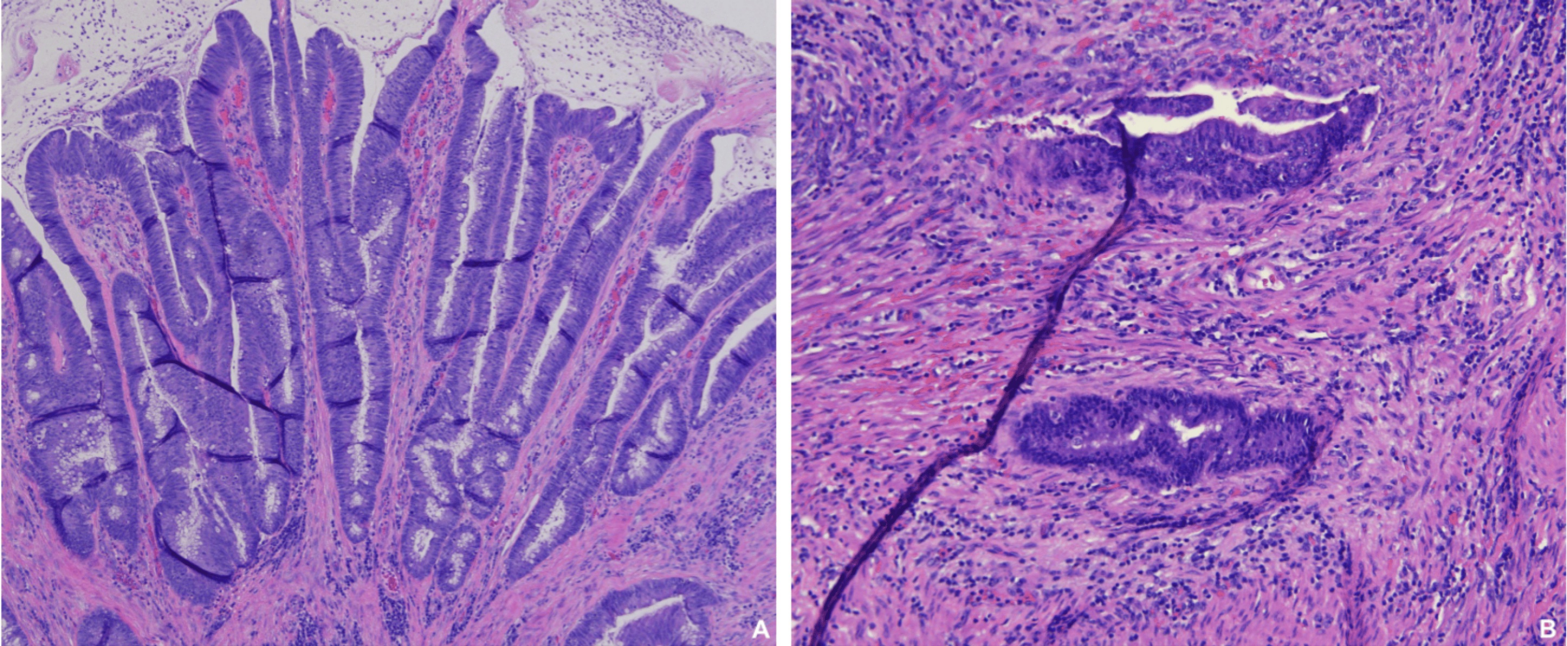
H&E-stained histopathology samples demonstrating villous adenoma tissue with high-grade dysplasia (A), and foci of pseudo-invasion (B).

**Figure 5:**
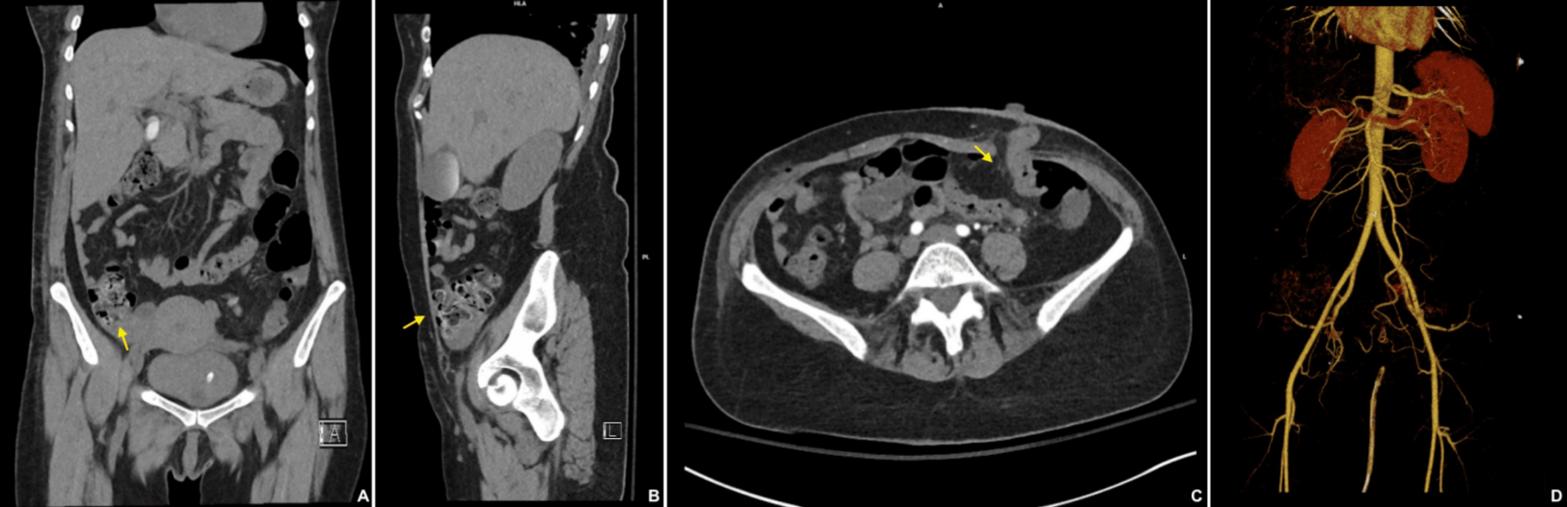
Post-operative computed tomography imaging of the abdomen and pelvis with contrast, demonstrating no evidence of active bleeding, including of the ostomy site (yellow arrows), and visualized small and large bowel. (A) Coronal view. (B) Sagittal view. (C) Transverse view. (D) Angiography.

Surgical pathology described an 8 x 6 x 4 cm unoriented aggregate of red polypoid mucosa (Figure 3), containing fragments of villous adenoma with extensive high-grade dysplasia, and foci of pseudo-invasion (Figure 4). No invasive carcinoma was identified throughout the entire specimen. The patient was seen by the Colorectal Surgery service on post-operative day one, who conducted a digital rectal exam and flexible sigmoidoscopy. Flexible sigmoidoscopy revealed numerous large, carpet-like polyps involving the rectum and extending close to the anal verge. The upper rectum and sigmoid colon appeared healthy, without any evidence of polyposis. The rectal wall was completely viable. Although some polyps showed a dusky surface coloration, rectal ischemia was not suspected. The patient required a further transfusion of 2 units of packed red blood cells. An interval CT scan did not demonstrate any evidence of active bleeding (Figure 5).

The patient was discharged on Hospital Day 11 after tolerating a regular diet, ambulating, and with adequate pain control and return of bowel function. Both the patient and her partner received education on ostomy care. She will continue to be followed by Colorectal Surgery for continued work-up and determination of definitive treatment.

Following discharge, repeat colonoscopy through the anus and colostomy site demonstrated carpet-like polyps restricted to the rectum. The patient then underwent a technically challenging transanal minimally invasive surgery (TAMIS), with further biopsy and removal of very friable rectal polyps. Pathology and MRI Pelvis findings following these procedures demonstrated evidence of invasive malignancy. The patient underwent flexible sigmoidoscopy, with biopsies collected utilizing cold forceps. Pathology results from these samples conflicted with prior findings, demonstrating tubulovillous adenoma with foci of high-grade dysplasia, without evidence of invasive carcinoma. As malignancy could not be confirmed, the patient did not receive any chemotherapy or radiation therapy.

Following multiple multi-disciplinary tumor board discussions, the patient was offered oncologic resection via abdominal perineal resection (APR) versus transanal transabdominal rectal resection (TATA) with hand-sewn super-low coloanal anastomosis. APR would result in the total removal of her sigmoid colon, rectum, and anus, whereas the TATA procedure would convert her loop colostomy into an ileostomy and preserve intestinal continuity. This procedure would not eliminate the risk of a permanent stoma, as full return of bowel control cannot be guaranteed. Further, the patient suffered the development of a severe distal rectal stricture following the TAMIS procedure, which has made it difficult to reliably monitor her rectum. The patient was referred to a tertiary/quaternary care referral center for a second opinion and further discussion regarding appropriate, conclusive treatment. 

## Discussion

There are a variety of conditions that place adult patients at risk for rectal prolapse, including chronic constipation or diarrhea, previous history of pelvic dysfunction or anatomical abnormalities, or the presence of other conditions that lead to increased intra-abdominal pressure [3]. In our case, constipation and the presence of rectal polyps predisposed the rectum to prolapse. Acutely prolapsed, reducible polyps may be treated with colonoscopic polypectomy. Villous adenomas confined to the rectal area may be eligible for transanal local excision. Lesions that are very large and sessile, those known to be an invasive malignancy, or those that are high risk for lymph node metastasis must be surgically resected for definitive treatment [4,5]. Regardless of presentation or histopathological findings, essential surgical considerations include the likelihood of treating incontinence, the chance of preventing recurrence, patient quality of life, and the presence of high-risk features such as fungation, ulceration, bowel wall distortion, or necrosis [4]. Risk factors for recurrence include polyp size, polyp number, and histology findings [6].

When manual reduction fails, sugar application can be attempted as a conservative, non-invasive method of triggering spontaneous reduction [7]. Should this fail, further attempts and exams under anesthesia may be considered. An irreducible prolapse can lead to severe complications, including pain, bleeding or hemorrhage, strangulation, or perforation [8]. Further studies - including, but not limited to, ultrasound, CT, or magnetic resonance imaging - can be utilized to rule out associated or predisposing conditions, as well as characterize the severity of the patient’s condition [3]. Imaging can also assist with determining surgical management. Colonoscopy and proctoscopy may also be performed both pre-operatively and at follow-up. Should work-up reveal evidence of necrosis, incarceration, or strangulation, surgical intervention is required to best preserve tissue and function, as well as prevent severe complications like infection, perforation, or worsening of the patient’s condition due to swelling or ischemia [9]. 

The optimal approach (abdominal versus perineal, laparoscopic versus open) must be individualized and account for patient characteristics and presenting symptoms, surgeon preference, and patient fit. Features of the rectal mass, including the presence and depth of invasion, size of the mass, and location in relation to anal verge, are also important determinants of surgical approach [10]. Abdominal approaches are associated with lower recurrence rates but higher postoperative morbidity. Perineal approaches, typically reserved for elderly patients or those with multiple comorbidities, are associated with higher recurrence rates but lower postoperative morbidity [7,11]. In cases of known tumors, preoperative chemotherapy may be beneficial for reducing tumor volume, increasing the likelihood of intact resection, and improving disease-free survival and anal function [12].

The most notable perineal technique is the perineal proctosigmoidectomy or “Altemeier procedure”. It is often the technique of choice for the treatment of full-thickness rectal prolapses, especially in instances of incarceration [13]. Given the emergent nature of our case, we resected mucosal lesions to reduce the prolapse and deferred further management to a Colorectal surgeon. Radical surgery incurs the risk of permanent colostomy, which has significant implications for a patient’s quality of life; however, this is often the safest management option given the risks associated with overlooking a possibly malignant lesion [14]. There is a benefit to repeat examinations and histopathological studies, with deeper biopsy sections and alternative staining, as was done in our case [15,16].

Histologically, villous adenomas comprise approximately 10% of colorectal adenomas. Yet, they carry a 35-40% risk of harboring malignancy. Adenomas greater than 2 cm confer a 46% risk [3,4]. Female sex also confers a minor increased risk of malignancy [6]. Complete excision of a polyp demonstrating high-grade dysplasia can be curative.

Management considerations in emergent presentations

Management of rectal prolapse is highly complex and nuanced. The goals of surgical management involve prolapse resolution and the correction of any associated functional abnormalities, including constipation or incontinence. Acute presentations and severe symptoms should generally be treated as anorectal emergencies necessitating immediate attention. In rare instances of incarceration, prompt surgical management prevents life-threatening or severe complications. Treatment and operative decisions should also be individualized to the patient and take into account the burden of illness, quality of life, and how emergent the patient’s presentation is. Patients with a history of anorectal adenomatous polyps are at increased risk of future malignancy, even with complete surgical resection. As such, surveillance and regular follow-up with a colorectal surgeon are recommended.

In the case of our patient, we elected to conduct a diverting colostomy given her history of chronic constipation, the extensive dissection required, as well as the likely need for future procedures. This procedure allowed for the prevention of prolapse and bleeding recurrence, while still permitting continued work-up, further perineal or rectal excisions, or radiation treatment, depending on the final diagnosis. Further, emergency medical services reported an estimated 500-1500 mL volume of blood loss while at home, and the patient required multiple blood transfusions prior to resection. Diverting the patient’s fecal stream via ostomy allowed for improved healing and immediate stabilization. Follow-up with Colorectal Surgery will discern between abdominal perineal resection versus transanal transabdominal rectal resection as definitive treatment.

## Conclusions

This is a case of a rare incarcerated rectal prolapse of polypoid tissue, in which the patient had very few risk factors or predisposing family history. Urgent surgical intervention was required to preserve mucosal tissue and prevent a multitude of other severe complications. Though these cases and presentations are uncommon, in the setting of incarcerated or strangulated rectal polyps, surgical intervention is still indicated.
